# Suppression of allograft rejection by regulatory B cells induced via TLR signaling

**DOI:** 10.1172/jci.insight.152213

**Published:** 2022-09-08

**Authors:** Kang Mi Lee, Qiang Fu, Guoli Huai, Kevin Deng, Ji Lei, Lisa Kojima, Divyansh Agarwal, Peter van Galen, Shoko Kimura, Naoki Tanimine, Laura Washburn, Heidi Yeh, Ali Naji, Charles G. Rickert, Christian LeGuern, James F. Markmann

**Affiliations:** 1 Center for Transplantation Sciences, Department of Surgery, Massachusetts General Hospital, Harvard Medical School, Boston, Massachusetts, USA.; 2 Organ Transplantation Center, Sichuan Provincial People’s Hospital and School of Medicine, University of Electronic Science and Technology of China, Chengdu, China.; 3 Division of Transplantation Surgery, Department of Surgery, Hospital of the University of Pennsylvania, Philadelphia, Pennsylvania, USA.; 4 Division of Hematology, Department of Medicine, Brigham and Women’s Hospital, Harvard Medical School, Boston, Massachusetts, USA.; 5 Division of Transplantation Surgery, Department of Surgery, Massachusetts General Hospital, Harvard Medical School, Boston, Massachusetts, USA.; 6 Department of Gastroenterological and Transplantation Surgery, Hiroshima University, Hiroshima, Japan.

**Keywords:** Immunology, Transplantation, Cellular immune response, Immunotherapy, Tolerance

## Abstract

B lymphocytes have long been recognized for their critical contributions to adaptive immunity, providing defense against pathogens through cognate antigen presentation to T cells and Ab production. More recently appreciated is that B cells are also integral in securing self-tolerance; this has led to interest in their therapeutic application to downregulate unwanted immune responses, such as transplant rejection. In this study, we found that PMA- and ionomycin-activated mouse B cells acquire regulatory properties following stimulation through TLR4/TLR9 receptors (Bregs-TLR). Bregs-TLR efficiently inhibited T cell proliferation in vitro and prevented allograft rejection. Unlike most reported Breg activities, the inhibition of alloimmune responses by Bregs-TLR relied on the expression of TGF-β and not IL-10. In vivo, Bregs-TLR interrupted donor-specific T cell expansion and induced Tregs in a TGF-β–dependent manner. RNA-Seq analyses corroborated the involvement of TGF-β pathways in Breg-TLR function, identified potential gene pathways implicated in preventing graft rejection, and suggested targets to foster Breg regulation.

## Graphical abstract

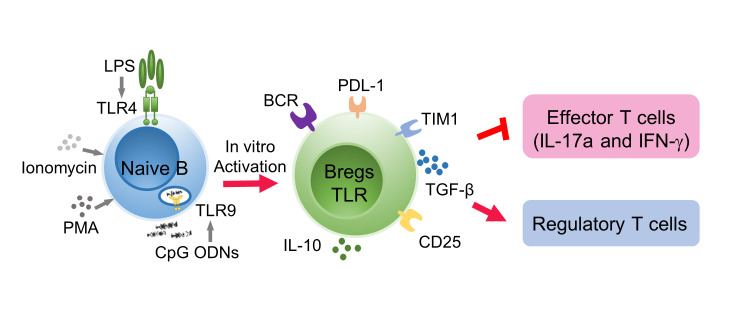

## Introduction

Achieving immunological tolerance to transplanted organs to avoid the morbidity associated with chronic immunosuppression remains an unmet need for the field of transplantation. Although efforts to achieve partial or full chimerism, such as by combining kidney and bone marrow transplants from the same living donor (1), have resulted in moderate success, this approach requires intensive pretransplant conditioning that precludes application to most transplant patients. An attractive alternative is the use of cell-based immunotherapies that can selectively abrogate donor-specific alloimmune responses. For example, Tregs have been evaluated in early phase clinical trials for their capacity to curtail alloimmunity and achieve allograft survival without chronic immunosuppression (2). Recently, Treg therapy trials in live donor kidney transplant recipients showed promise at immunosuppression minimization with a concomitant reduction in infectious complications 2 years after transplant (3). Likewise, a Treg trial in liver allograft recipients achieved rejection-free, immunosuppression-free transplant survival in 6 of 10 patients for over 5 years (4).

Although the immunomodulatory property of Tregs is now well established, the regulatory potential, if any, of other cell types has remained elusive. Although B lymphocyte–mediated suppression of cellular immunity was first noted in the 1970s (5, 6), more recent work has discovered a subpopulation of B cells with regulatory properties (Bregs) that can modulate immune response in a variety of oncologic, autoimmune, and inflammatory conditions (7, 8). A regulatory role for B cells in transplant tolerance has also been implicated in a nonhuman primate model of islet transplantation (9). In addition, studies on spontaneously tolerant human recipients of renal allografts have found substantial differences in the transcriptional profile and phenotype of B cells from peripheral blood compared with those recipients still on immunosuppression (10). Collectively, these data suggest an active role for Bregs in transplant tolerance induction. Unlike Tregs that can be defined by expression of the master transcriptional regulator Foxp3, there is a paucity of lineage-specific identifiers for Bregs (11, 12). To date, the most widely utilized marker of Breg activity has been the expression/secretion of IL-10, which is also a key effector molecule conveying regulatory activity. As evidence of the functional heterogeneity of the Breg population, B cell–mediated regulation has also been associated with the expression of TGF-β, programmed cell death ligand 1 (PD-L1), and granzyme B.

As proof of concept for the potential of Bregs as a cell-based therapy, the current study demonstrates that naive B cells activated in vitro through TLR signaling are able to fully suppress T cell proliferation in vitro and promote allograft tolerance in mice. Through extensive in vivo assessment of graft-reactive T cell proliferation, we identify that Breg-mediated suppression is TGF-β dependent. We further analyzed the cellular intricacies of Breg subsets engaged in dampening alloreactive responses using single-cell RNA-Seq (scRNA-Seq). These analyses further revealed that suppressive B cell subpopulations are characterized by low levels of MHC class I and class II and B cell receptor (BCR) gene expression, together with substantial enrichment and activity of the TGF-β and IL-2/STAT5 signaling pathways.

## Results

### Phenotypic changes of B cells through TLR-mediated activation.

The population of donor-specific Bregs from mice with Ab-induced transplant tolerance is small (13, 14). Thus, we developed a protocol to expand B cells through in vitro TLR signaling with PMA/ionomycin and discovered that this treatment was sufficient to engender regulatory activity. We studied 3 distinct B cell populations, all derived from enriched splenic B cells from C57BL/6 (B6) mice: naive B6 B cells (B cells-naive), B cells stimulated in vitro by the TLR9 agonist CpG ODN 1668 for 3 days (Bregs-CpG), and B cells stimulated by CpG for 3 days with the addition of the TLR4 agonist LPS, PMA, and ionomycin (LPI) for the last 5 hours of culture (Bregs-TLR).

**Figure 1:**
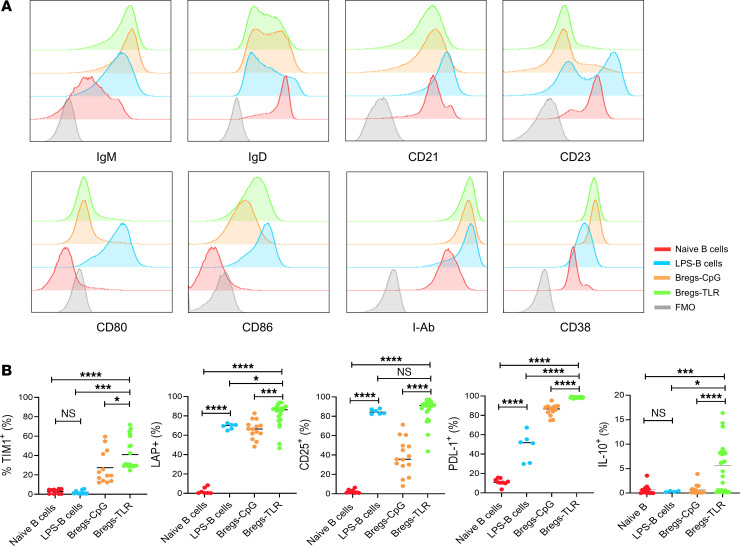
Phenotypic characterization of TLR-activated B cells. (**A**) Purified splenic naive B cells were stimulated with the TLR9 agonist CpG (ODN 1668) for 3 days (Bregs-CpG) and with LPS, PMA, and ionomycin added for the last 5 hours (Bregs-TLR). Cell subsets are naive B cells (red), LPS-B cells (blue), Bregs-CpG (orange), Bregs-TLR (green), and control fluorescence minus one (FMO) (gray). Cell subsets were analyzed for expression of IgM, IgD, CD21, CD23, CD38, CD80, CD86, and MHC class II (I-Ab). Data are representative of independent experiments performed at least 3 times. (**B**) Flow cytometry analysis of regulatory B cell–associated markers. Frequency (% of positive cells/CD19^+^ B cells) of TIM1^+^, LAP^+^, CD25^+^, PD-L1^+^, and IL-10^+^ B cells (≥ 5 independent experiments). Data are expressed as mean. *P* values (1-way ANOVA): *P < 0.05, ***P < 0.001, and ****P < 0.0001. TIM1, T cell immunoglobin domain and mucin domain protein 1; LAP, latency-associated peptide.

Compared to B cells-naive, both Bregs-CpG and Bregs-TLR manifest phenotypic changes in IgM^hi^IgD^intermediate/lo^ expression, suggesting an immature phenotype. The CD21 and CD23 staining is suggestive of a phenotypic similarity to immature transitional B cells (IgM^+^IgD^–^CD21^–^CD23^–^) and marginal zone B cells (CD19^+^CD21^hi^CD23^lo^), which have been previously shown to exhibit regulatory function (15) (Figure 1A). Bregs-TLR and Bregs-CpG similarly upregulated markers of B cell activation including CD80, CD86, MHC class II (I-Ab), and CD38 (Figure 1, A and B) (16). Bregs-TLR and Bregs-CpG differed, however, in that the former manifested significantly greater expression of TIM1, LAP (a surrogate marker for TGF-β expression), CD25, and PD-L1^+^ (Figure 1B). Of note, Ab-induced Bregs are known to promote islet graft acceptance through in vivo induction of Tregs via a TGF-β–dependent mechanism (17, 18). B cell suppressive function has also been related to cytotoxic activity via PD-L1 (killer B cells) (19). Contrary to the previous report (20), Bregs-CpG and Bregs-TLR cell subsets contained few CD138^+^ plasmablasts (not shown), likely a consequence of different activation conditions (100 nM/200 μL CpG ODN 1668 for 7 days vs. 1.6 nM/200 μL for 3 days in our model).

### Bregs-TLR suppress effector T cell proliferation and prevent rejection of allogeneic grafts.

**Figure 2:**
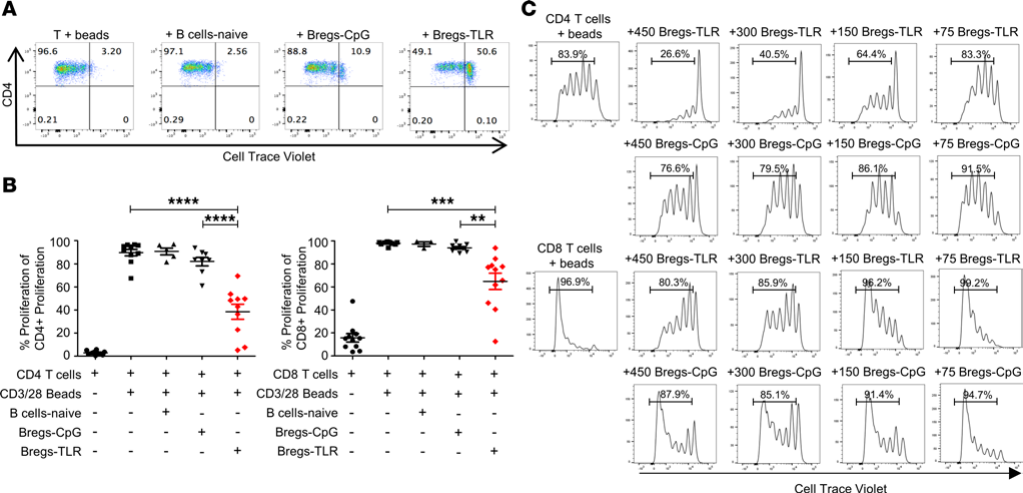
Bregs-TLR suppress in vitro T cell proliferation in a dose-dependent manner. (**A**) Representative flow cytometry plots of suppression assays: effector cells were purified splenic CD4^+^ T cells from B6 mice labeled with CellTrace Violet (CTV) and stimulated by anti-CD3/CD28 beads (beads); suppressor cells were naive B cells, Bregs-CpG, or Bregs-TLR. Suppression assays were run for 4 days. (**B**) Cumulative data of CD4^+^ and CD8^+^ T cell proliferation in 4-day suppression assays. *P* values (1-way ANOVA): **P < 0.01, ***P < 0.001, and ****P < 0.0001. (**C**) Proliferation histograms of CD3/CD8-activated and CTV-labeled CD4^+^ (top 2 rows) or CD8^+^ T cells (bottom 2 rows, 150,000 cells/assay), cocultured for 4 days with increasing numbers (75, 150, 300, and 450K) of Bregs-TLR or control Bregs-CpG.

Next, we explored the ability of Bregs-CpG and Bregs-TLR to suppress in vitro proliferation of purified T cells. Using a CD3-CD28 bead stimulation assay, we found that Bregs-TLR, but not Bregs-CpG, potently suppressed both CD4^+^ and CD8^+^ T cell proliferation compared with B cells-naive (Figure 2, A and B). Furthermore, the suppression by Bregs-TLR was dose-dependent for both CD4^+^ and CD8^+^ T cells (Figure 2C).

**Figure 3:**
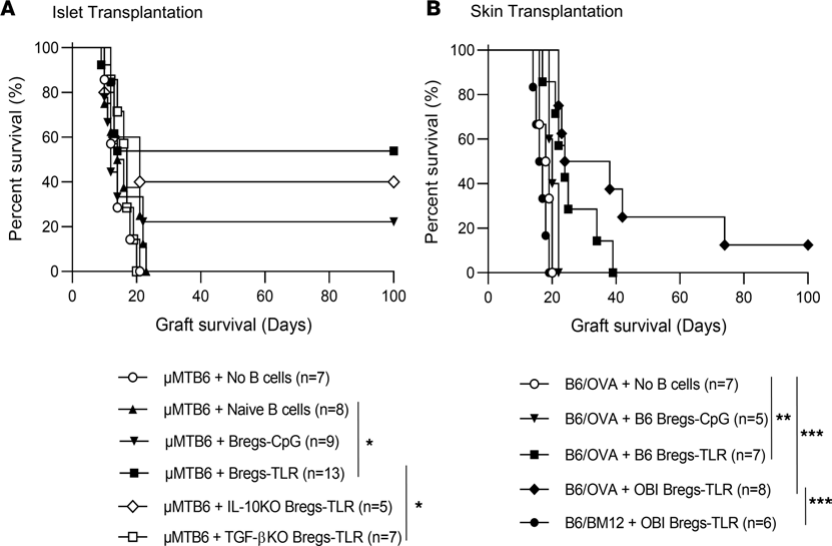
Bregs-TLR confer increased graft survival. (**A**) Survival of BALB/c (H-2^d^) islet allografts (Kaplan-Meier plot) in μMT B6 recipient mice (H-2^b^) induced by adoptive transfers of various B cell subsets. Purified CD19^+^ B cells tested were naive B cells, Bregs-CpG, and Bregs-TLR derived from WT B6, IL-10–deficient, or TGF-β–deficient (KO) mouse strains. (**B**) OVA^+^ skin graft survival following adoptive transfer of Bregs-CpG or Bregs-TLR. Specificity controls included Bregs-TLR derived from B6 or anti-OVA transgenic BCR OBI as well as graft control B6/BM12 that expresses a transplantation antigen unrelated to OVA. *P* values indicate log-rank test. *P < 0.05, **P < 0.01, and ***P < 0.001.

To further assess the in vivo regulatory function of Bregs-TLR, this cell population was adoptively transferred to diabetic B cell–deficient μMT mice transplanted with allogeneic BALB/c islets. Recipients were either untreated or injected i.v. with 5 × 10^6^ B cells-naive, Bregs-CpG, or Bregs-TLR and followed for islet allograft survival by serial blood glucose measurements. We observed that transferred Bregs-TLR promoted allograft survival with 7 out of 13 recipients achieving graft survival for more than 100 days, while mice receiving no B cells or naive B cells all lost graft function by 23 days with a median survival time (MST) of 14 days (no B cells vs. Bregs-TLR, *P* = 0.042) and 15 days (B cells-naive vs. Bregs-TLR, *P* = 0.041), respectively (Bregs-CpG MST 13 days vs. Bregs-TLR undefined MST, *P* = 0.069) (Figure 3A). Bregs-TLR significantly prolonged islet allograft survival, with approximately 54% of recipients achieving long-term survival compared with approximately 17% by Bregs-CpG (Figure 3A). To further validate the contribution of Bregs-TLR–derived IL-10 and TGF-β in suppressing allograft rejection, we generated Bregs from mice with B cells deficient in IL-10 (from IL-10^–/–^ mice) or TGF-β (from CD19^cre/WT^ TGF-β^fl/fl^ mice) (21). BALB/c islets were transplanted to μMT recipients, which were also injected with either WT, TGF-β^–/–^, or IL-10^–/–^ Bregs-TLR cells. Recipients of TGF-β^–/–^ Bregs-TLR cells rejected all allografts within 20 days after transplant, whereas 2 of the 5 recipients treated with IL-10^–/–^ Bregs-TLR demonstrated long-term graft survival similar to WT Bregs-TLR (Figure 3A). These results suggest that TGF-β secretion from Bregs-TLR is essential to prevent or delay allograft rejection.

We also evaluated skin graft survival using an antigen-specific model of graft rejection, transplanting skin to B6 recipients from donor OVA^+^ B6 mice that ubiquitously express OVA in all tissues. OVA^+^ skin grafts were promptly rejected by recipients with both no B cell transfer and with Bregs-CpG transferred with an MST of 18.5 and 20 days, respectively. In contrast, OVA skin graft survival was extended by adoptive transfer of B6 Bregs-TLR to an MST of 24 days (no B cells vs. B6 Bregs-TLR, *P* = 0.0041; Bregs-CpG vs. B6 Bregs, *P* = 0.069) (Figure 3B). OBI Bregs-TLR derived from OBI mice, which have a clonally restricted B cell repertoire specific for OVA, achieved the longest survival with an MST of 31 days (no B cells vs. OBI Bregs-TLR, *P* = 3.0 × 10^4^).

### Role of Bregs-TLR in the control of antigraft CD4 and CD8 responses.

**Figure 4:**
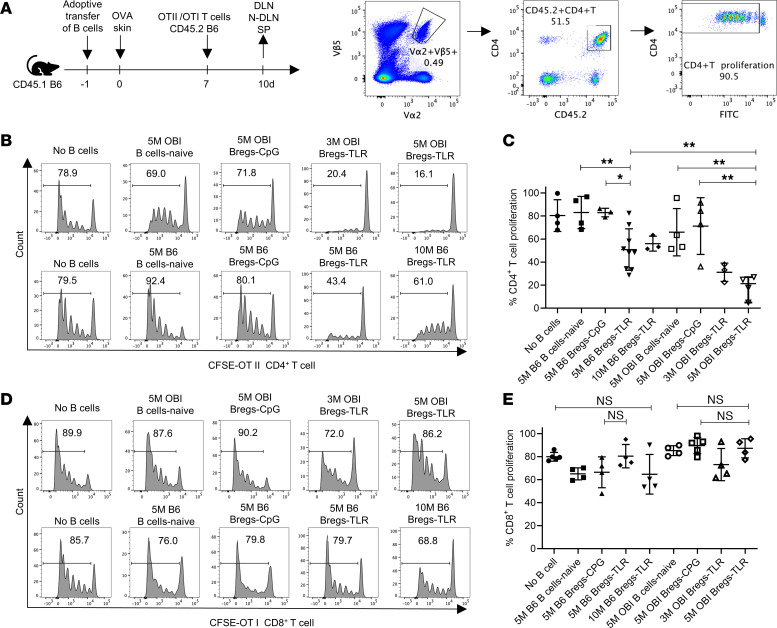
Role of Breg BCR specificity in suppression of T cell proliferation. (**A**) Experimental design: Graft recipients were CD45.1 B6 mice; cells transferred at day –1 were Bregs-TLR or naive B cells from OBI/B6 (CD45.2); and grafting of OVA^+^ skin was done at day 0. After 7 days, 3–10 × 10^6^ (3–10M) CFSE-labeled OTII or CTV-labeled OTI anti-OVA T cells were injected i.v. Analysis of T cell proliferation was done by day 10 on cells from DLNs (inguinal, axillary, and branchial lymph nodes ipsilateral to the skin graft), N-DLNs (contralateral), and spleen (SP). Gating strategy: inhibition of OVA-specific CD4^+^ T cells by Bregs-TLR was further evaluated by FACS gating on CD4^+^Va2^+^Vb5^+^CD45.2^+^ T cells. (**B** and **C**) Frequency of CD4^+^ T cell proliferation (% proliferating CD4^+^/total CD4^+^ T cells) in host DLNs harvested after injection of Bregs-TLR or Bregs-CpG derived from OBI (top row) or B6 mice (bottom row). (**D** and **E**) Same experimental design as in **B** and **C**, applied to OTI CD8^+^ T cells. Number of mice in each group was greater than or equal to 3. *P* values (1-way ANOVA): *P < 0.05 and **P < 0.01.

The function of Bregs may be sensitive to their microenvironment or tissue context. Given the encouraging observations from the aforementioned in vitro assays, we pursued an in vivo approach to better delineate the mechanistic underpinnings of Bregs-TLR function. The experiment focus was to evaluate the effects of Bregs on graft reactive CD4^+^ and CD8^+^ T cells. An antigen-defined model of graft rejection was used using lymphocytes from B6 background mice with transgene-encoded receptor specificity for OVA (OTI CD8^+^ T cells, OTII CD4^+^ T cells, and OBI B cells). In addition, skin grafts were syngeneic to hosts, except for the expression of OVA (B6-OVA) (22–24). CD45.1^+^ B6 mice were first injected with purified CD45.2^+^, CTV-labeled OTI, or CFSE-labeled OTII T cells with/without Bregs-TLR generated from OBI mice (OBI Bregs-TLR). Cell recipients were also transplanted with B6-OVA skin according to the timeline detailed in Figure 4A. Responses from OVA-specific T cells were evaluated in the graft’s draining lymph nodes (DLNs) and contralateral (nondraining) lymph nodes (N-DLNs). The spleen, DLNs, and N-DLNs were harvested to measure the Bregs’ impact on OTI and OTII T cell proliferation 10 days after graft placement (Figure 4, A–E). In this model, donor-derived passenger leukocytes from OVA skin traffic to secondary lymphoid tissues of the recipient and the donor antigens are processed and presented to reactive OTI and OTII T cells (25, 26).

The impact of Bregs on OTII CD4^+^ cell proliferation in vivo is presented in Figure 4, B and C, which revealed that 80.4% ± 13.8% of OTII cells proliferated. Similar OTII proliferation was seen in recipients of control B6 Bregs-CpG cells (83.0% ± 3.7%). However, following administration of 5 × 10^6^ B6 Bregs-TLR, OTII T cell proliferation fell to 50.9% ± 18.1% (*P* < 0.05 vs. no B cells). When compared with B6 Bregs-TLR, OVA-specific OBI Bregs-TLR demonstrated stronger suppression in a dose-dependent manner: from 31.2% ± 7.9% (*P* < 0.01) for 3 × 10^6^ OBI Bregs-TLR, down to 18.6% ± 9.9% versus (*P* < 0.01) for 5 × 10^6^ OBI Bregs (Figure 4, B and C). These data imply that Bregs with specificity to cognate graft antigen (OVA) downmodulate CD4^+^ effector proliferation more efficiently than Bregs with polyclonal BCR specificities. The preactivation of OBI Bregs-TLR by graft antigen may augment the degree of suppression of these cells, an eventuality that will be discussed later.

As reported in Figure 4, D and E, none of the B6 Bregs-TLR or OBI Bregs-TLR doses tested had any significant effect on decreasing CD8^+^ OTI cell proliferation in vivo. Interestingly, CD4^+^ T cells and CD8^+^ T cells demonstrated the highest proliferative activity in different anatomic locations in this model. For CD4^+^ T cells, donor-reactive proliferation was largely restricted to the DLNs, with minimal proliferation in N-DLNs (*P* < 0.001 vs. DLNs) and spleen (*P* < 0.0001 vs. DLNs). In contrast, CD8^+^ T cell proliferation was evident in the DLNs and spleen, but at lower levels in the N-DLNs (*P* < 0.001 vs. DLNs).

### OBI Bregs-TLR convert CD4^+^CD25^–^ T cells to CD4^+^CD25^+^Foxp3^+^ Tregs through TGF-β.

**Figure 5:**
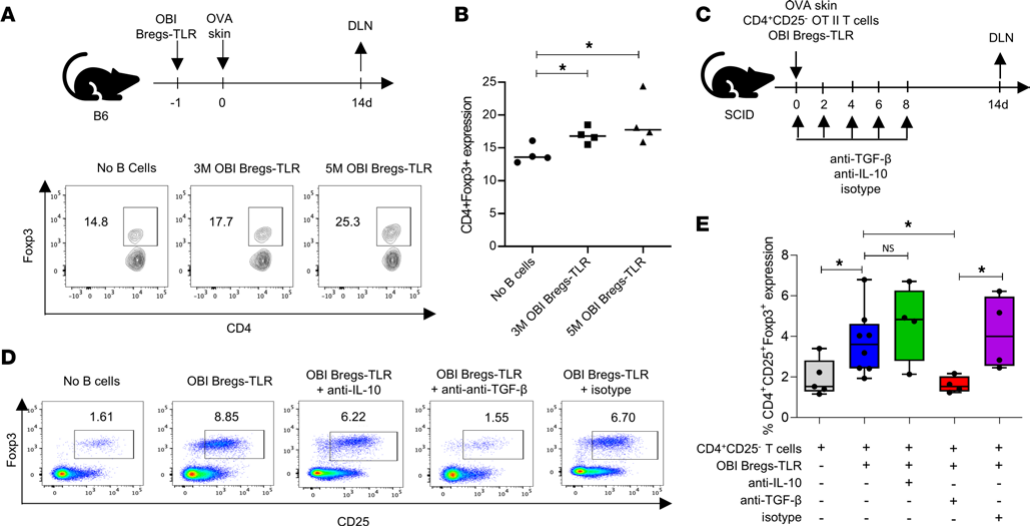
Bregs-TLR convert CD4^+^CD25^–^ T cells into CD4^+^CD25^+^Foxp3^+^ Tregs in a TGF-β–dependent manner. (**A**) Experimental plan and representative contour plot of DLN CD4^+^Foxp3^+^ Tregs (from CD45.1 hosts), 14 days after Breg infusion (3M or 5M) and OVA skin transplantation (*n* = 4). (**B**) Scatter dot plot analysis (mean ± SD) of Treg expansion. (**C**) T cell–deficient SCID mice transplanted with OVA skin received the same day 5M OB1 Bregs-TLR and 1M CD4^+^CD25^–^ OTII T cells. Ab treatments were done as indicated with anti–IL-10 (250 μg/injection) or anti–TGF-β (200 μg/injection). Treg analysis was performed 14 days after transplantation on groups of at least 4 mice. (**D**) Representative FACS dot plots showing the conversion of CD4^+^CD25^–^ OTII T cells into CD4^+^CD25^+^Foxp3^+^ Tregs. (**E**) Treg conversion in various groups: box-and-whisker plot analysis showing means (horizontal lines) and range of values (vertical bars). *P* value (1-way ANOVA): *P < 0.05.

Our previous work showed that Bregs, generated via the IL-4 and TIM1 pathways, did prolong allograft survival at least in part through the induction of Tregs (13, 17). Given that Bregs-TLR prolong graft survival and suppress CD4^+^ T cell proliferation in vivo, we assessed whether Bregs-TLR–induced graft prolongation also correlated with Treg induction. Using the OVA skin graft model on WT B6 recipients, we examined the DLNs, as depicted in Figure 5, A and B, for the presence of CD4^+^Foxp3^+^ Tregs. We observed that without any treatment, the proportion of Tregs was 14.0% ± 1.4% of total CD4^+^ cells; numbers that increased to 16.9% ± 1.2% to reach 19.0% ± 3.7% (*P* < 0.05 vs. no B cells), following administration of 3 × 10^6^ or 5 × 10^6^ OBI Bregs-TLR, respectively (Figure 5, A and B).

Based on evidence that Bregs induce Tregs through TGF-β but not IL-10 (27, 28), and in accordance with our observation on high expression of TGF-β in Bregs-TLR (Figure 1), we investigated the role of TGF-β in Treg induction. Sorted CD4^+^CD25^–^ effector T cells from OTII mice were adoptively transferred to severe combined immunodeficient (SCID) recipient mice grafted with OVA skin (Figure 5C). On the same day, 5 × 10^6^ OBI Bregs-TLR or control naive OBI B cells were injected with or without anti–TGF-β Ab to neutralize TGF-β in vivo (17, 24). We found that the proportion of CD4^+^CD25^–^Foxp3^–^ OTII T cells transferred to SCID mice transitioned to CD4^+^CD25^+^Foxp3^+^ Tregs around day 14 at a significantly higher level in mice cotransferred with OBI TLR-Bregs (3.7% ± 1.6%) compared with controls (1.9% ± 0.9%) (*P* < 0.05 vs. no B cells) (Figure 5, D and E). Furthermore, when the OBI Bregs-TLR treatment group was also conditioned with the anti–TGF-β Ab, the proportion of CD4^+^CD25^+^Foxp3^+^ Tregs decreased by more than 2-fold (1.6% ± 0.4%, *P* < 0.05, vs. OBI Bregs-TLR). In contrast, the proportion of Foxp3^+^ T cells was not significantly reduced after IL-10 neutralization (4.6 % ± 1.9%, *P* > 0.5 vs. OBI Bregs-TLR), similar to the results obtained with TGF-β isotype control (4.2% ± 1.8%) (Figure 5, D and E). Collectively, these data indicate that OBI Bregs-TLR cells promote the conversion of a significant fraction of CD4^+^CD25^–^ effector cells toward the CD4^+^CD25^+^Foxp3^+^ Treg phenotype. This occurs in a TGF-β–dependent but IL-10–independent manner. Further attempts to evaluate the role of Tregs in Bregs-TLR suppression, through in vivo Treg depletion in the FoxP3^DTR^ mouse model, were unsuccessful due to the previously described occurrence of autoimmune conditions that compromised graft survival (29).

### Control of suppressive Bregs on cytokine-producing T cells.

**Figure 6:**
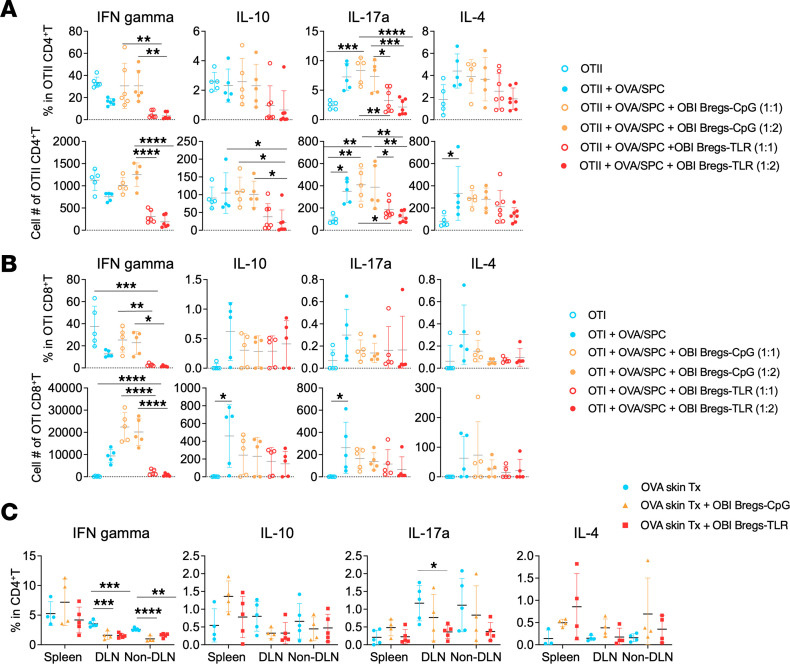
Impact of suppressive Bregs on cytokine-producing T cells. In vitro frequencies and numbers of cytokine-producing T cells in cocultures of OTII CD4^+^ (**A**) or OTI CD8^+^ T cells (**B**) stimulated by irradiated OVA splenocytes (OVA/SPC) and incubated for 3 days with OVA-specific OBI Bregs-TLR or control OBI Bregs-CpG cells (T cells: Bregs ratios of 1:1 and 1:2). (**C**) B cell–deficient μMT B6 mice were injected with purified 1 × 10^6^ OTII CD4^+^ T cells and 5 × 10^6^ Bregs-TLR or Bregs-CpG at day –1. Transplantation of OVA skin grafts was done at day 0, and the in vivo frequencies of OTII T cells producing cytokines were evaluated at day 14 (≥ 4 independent experiments). Data are expressed as mean ± SD. *P* value (1-way ANOVA): *P < 0.05, **P < 0.01, ***P < 0.001, and ****P < 0.0001.

Experiments were performed using OBI B cells (with a transgenic BCR anti-OVA) to derive OB1 Bregs-TLR/CpG. In vitro studies involved cocultures of anti-OVA CD4^+^ (OT-II) or CD8^+^ (OT-I) T cells stimulated by irradiated OVA splenocytes with OBI Bregs-TLR or control OBI Bregs-CpG (Figure 6, A and B). In vivo settings included concomitant injection of OTII and Bregs in B cell–deficient μMT mice 1 day prior to OVA skin transplantation (Figure 6C). In both experimental designs, frequencies of cytokine-producing cells were determined by FACS analysis of intracellular staining done 14 days after transplantation. As seen in Figure 6A, Bregs-TLR suppression acted by significantly decreasing the frequencies and numbers of CD4^+^ T cells expressing IFN-γ and IL-17a. The amount of cytokine detected per cell was not affected as the MFI remained comparable to that of controls (not shown). Bregs-TLR had no effect on CD4^+^ T cell expression of IL-10 or IL-4. These results were corroborated in vivo (Figure 6C). Similar studies, implemented in models of B6 T cells responding to BALB/c allogeneic targets in vitro, confirmed the initial observations obtained in the OVA model. As our studies have examined the cytokine status of both suppressive Bregs and suppressed effector T cells, it may be valuable to summarize our findings: no other cytokines than IL-10 and TGF-β showed differential expression between nonsuppressive Bregs-CpG and suppressive Bregs-TLR (Figure 1). Likewise, the effects of Bregs-TLR suppression on cytokine-producing T cells were attested by a significant reduction of T cells expressing IFN-γ and/or IL-17a (Figure 6).

### Bregs-TLR are highly heterogeneous in phenotype and functions.

**Figure 7:**
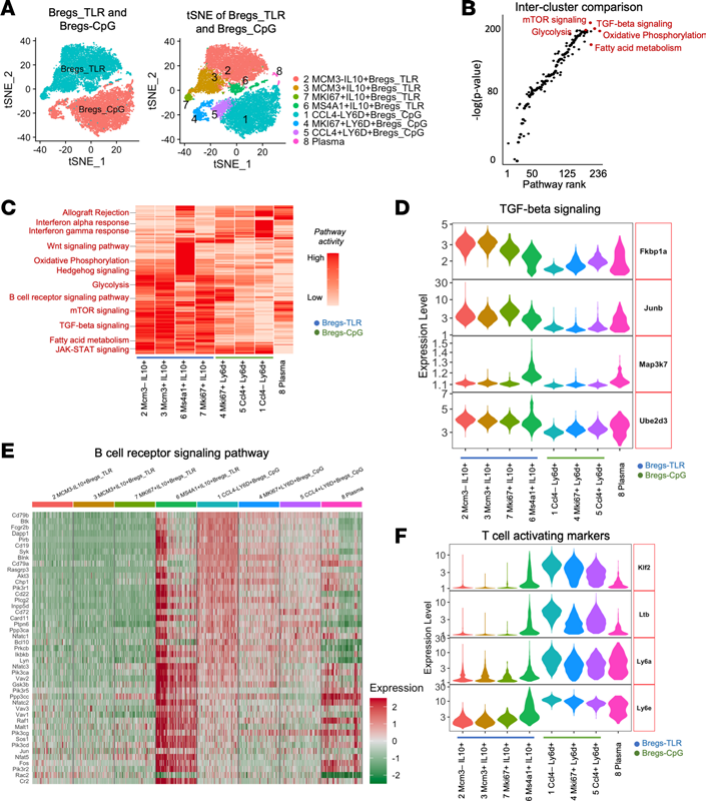
Phenotyping of Bregs-TLR by scRNA-Seq. (**A**) Comparative library and cluster analyses between Bregs-TLR and Bregs-CpG cells. Bregs-CpG cells are mainly distributed in 3 subsets (1, 4, and 5) characterized by the *CCL4^–^LY6D^+^*, *MKI67^+^LY6D^+^*, and *CCL4^+^LY6D^+^* phenotypes, respectively. The 4 main Bregs-TLR clusters (2, 3, 6, and 7) are defined by the *MCM3^–^IL10^+^*, *MCM3^+^IL10^+^*, *MS4A1^+^IL10^+^*, and *MKI67^+^MCM3^+^IL10^+^* phenotypes. Cells in cluster 8 carried markers of plasma cells. (**B**) Intercluster (8 clusters) pathway enrichment; top 5 signaling pathways enriched in Bregs-TLR are indicated. (**C**) Heatmap of gene pathways enriched in each cluster; top 12 Bregs-TLR–associated pathway signatures are displayed, including that of TGF-β signaling. (**D**) Cluster distribution of expression of genes involved in TGF-β signaling (violin plots). (**E**) Heatmap of cluster distinctive enrichment of genes implicated in BCR signaling. (**F**) Gene signatures downmodulated in Breg-TLR clusters 2, 3, and 7 but not in control Bregs-CpG (clusters 1, 4, and 5).

Finally, scRNA-Seq was performed to delineate the phenotype of Bregs-TLR and Bregs-CpG cells and potentially identify gene signatures characteristic of each Breg subset. A total of 8 clusters were analyzed for Breg subsets when the resolution was 0.3 (Supplemental Figure 1; supplemental material available online with this article; https://doi.org/10.1172/jci.insight.152213DS1). As shown in Figure 7A and Supplemental Figure 2A, Bregs-CpG mainly contained 3 clusters (showed by cell numbers and proportions): cluster 1 (7550/19,616, 38.49%), cluster 4 (913/19,616, 4.65%), and cluster 5 (912/19,616, 4.65%) corresponding to 1) *CCL4^–^LY6D^+^*, 4) *CCL4^+^LY6D^+^*, and 5) *MKI67^+^LY6D^+^*. Bregs-TLR clustered into 4 major subsets: cluster 2 (6749/19,616, 34.41%), cluster 3 (2177/19,616, 11.10%), cluster 6 (851/19,616, 4.34%), and cluster 7 (298/19,616, 1.52%). These subsets were annotated as 2) *MCM3^–^IL-10^+^*, 3) *MCM3^+^IL-10^+^*, 6) *MS4A1^+^IL-10^+^*, and 7) *MKI67^+^MCM3^+^IL-10^+^* Bregs-TLR (Figure 7A). Notably, a fraction of Bregs-CpG cells were also involved in cluster 6 (MS4A1^+^IL-10^+^ Bregs-TLR). Some Bregs-CpG and Bregs-TLR differentiated into *JCHAIN^hi^IGHM^hi^* plasma cells in cluster 8 (166/19,616, 0.85%). Relevant gene expression markers are shown in Supplemental Figure 2A.

To understand the functional features of these subpopulations, downstream pathway analysis identified more than 20 pathways positively enriched in Bregs-TLR (Figure 7, B and C, and Supplemental Figure 2B). Some enriched pathways, such as protein secretion, unfolded protein response, and TNF-α signaling via NF-κB, were specific to Bregs-TLR, whereas rejection-associated pathways, such as allograft rejection and IFN-γ and IFN-α responses, were Bregs-CpG specific. Additionally, more metabolism-relevant pathways were enriched in Bregs-TLR, suggesting a more highly activated state or metabolic demand in Bregs-TLR compared with Bregs-CpG (Figure 7C; Supplemental Figure 2, B and C; and Supplemental Figure 3, A and B).

As anticipated, the TGF-β signaling pathway was enriched in the prominent Bregs-TLR cluster 2, supporting initial observations (Figures 1 and 5). Key genes in downstream TGF-β signaling, such as *FKBP1A* (FK506-binding protein 1A), *MAP3K7* (TGF-β–activated kinase 1), *JUNB*, *UBE2D3* (ubiquitin-conjugating enzyme E2 D3), and other TGF-β–relevant genes, were significantly upregulated in Bregs-TLR (Figure 7D and Supplemental Figure 4). Expression of genes involved in IL-2/STAT5 signaling was likewise increased in Bregs-TLR subsets: clusters 2, 3, and 7, and plasma cells (cluster 8). In contrast, both IFN-γ response and IFN-γ–relevant key genes such as *IRF9* and *SAMHD1* and allograft rejection-relevant genes such as *CD2* and *FCGR2B* were positively enriched in Bregs-CpG compared with Bregs-TLR (Supplemental Figure 2, B and C). More importantly, both MHC class I and II–relevant pathways and MHC-relevant genes such as *H2-Oa*, *H2-Ob*, *H2-Ab1*, *H2-Aa*, *PIRB*, *CTSH*, and *MR1* were negatively enriched in Bregs-TLR subsets 2 and 3 (Supplemental Figure 5, A and B). Additionally, while BCR signaling and BCR-relevant genes such as *CD19*, *CD79a*, and *CD79b* were positively enriched in Bregs-CpG subsets 1, 4, and 5 (Figure 7E), T cell activation genes such as *LY6A*, *LY6D*, *LY6E*, *KLF2*, and *LTB* were upregulated in Bregs-TLR (Figure 7F). Collectively, gene signatures would characterize Breg-TLR cells as highly metabolically active cells that have upregulated gene pathways involved in immune regulation (TGF-β, IL-2/STAT5, and IL-10) while downregulating pathways are associated with effector function (MHC, IFN-γ, and BCR signaling). These studies have further substantiated an important role for TGF-β–mediated signaling in Bregs-TLR functions. On the opposite side of the spectrum, it appears that Bregs-CpG exhibit the phenotypes of activated effector B cells.

## Discussion

Here we report a simple method to generate Bregs by activation through the TLR9 and TLR4 receptors and stimulation with PMA and ionomycin. The requirement of each element of the stimulatory cocktail (LPI), added during the last 5 hours of culture, was tested in additional experiments. These showed that the development in vitro of suppressive Bregs-TLR cells requires PMA and ionomycin independently of LPS (not shown). The benefit of PMA/ionomycin stimulation in this Breg protocol would correlate with studies showing that B cells from tolerant patients manifested regulatory properties when stimulated with PMA/ionomycin (10). B cells activated in this manner manifest phenotypic changes while exhibiting potent suppression of alloimmunity in vitro and in vivo (Figures 2 and 3). In vitro, Bregs-TLR were more effective than B cells activated by TLR9 alone (Bregs-CpG) at TGF-β–dependent suppression of CD4^+^ and CD8^+^ T cell proliferation (Figure 2). In vivo Bregs-TLR inhibited the proliferation of donor-reactive CD4^+^ T cells in draining lymph nodes, promoted the generation of Tregs, and significantly extended islet and skin graft survival compared with Bregs-CpG or naive B cells (Figures 2–5). Breg-TLR populations with transgene-encoded specificity for a defined donor antigen demonstrated more potent suppression of donor-reactive T cells and increased duration of allograft survival as compared with polyclonal Bregs-TLR (Figure 3). These data highlight the importance of Breg preactivation prior to suppression and suggest that stimulation of donor-reactive Bregs-TLR via BCR provides optimal conditions for suppressive function. scRNA-Seq analysis both supports the relevance of TGF-β in securing graft survival and provides new insights into other key molecular pathways distinguishing potently suppressive Bregs-TLR from weakly suppressive Bregs-CpG. The ease with which Bregs-TLR can be generated in vitro, with predefined BCR specificity, advocates for the relevant translational potential of this approach.

It is noteworthy that although Bregs-TLR alone without other modifications conferred long-term graft survival in most recipients, delayed rejection did occur in a subset of animals (Figure 3). Reproducibility of i.v. injections of large numbers of Bregs together with poor suppression of CD8^+^ T cell proliferation by Bregs-TLR (Figure 2; 27% vs. 88% of suppression for CD8 vs. CD4) may in part explain incomplete graft protection. The main causes of graft rejection by some recipient mice remains under investigation. The inefficient suppression of graft-specific CD8^+^ T cell proliferation was likewise observed in vivo (Figure 4), a feature that may be due to a prompt in situ activation of OTI cells (high-affinity TCR for cognate OVA peptide) that cannot be tamed by Bregs arriving afterward on the graft site. By analogy to Tregs that have been shown to modulate OTI CD8^+^ effector differentiation in vivo by controlling IL-2 homeostasis (30), one could speculate that Bregs would not suppress CD8^+^ T cell proliferation but rather prevent their expansion by controlling cytokine homeostasis.

Based on our data and in accordance with the literature (24, 31), we surmise that the regulatory property of Bregs-TLR emerges from (i) donor antigen capture via the BCR, (ii) presentation of processed antigens to other regulatory cell types such as NK cells and Tregs, and (iii) secretion of immunomodulatory factors. Such hypotheses are founded on a number of observations: i) Bregs-TLR upregulate cell surface expression of MHC class II expression and other key costimulatory molecules such as CD80 and CD86 (Figure 1); ii) Bregs-TLR cells secrete several immunomodulatory cytokines including IL-10 and TGF-β and promote Treg generation (Figure 1); iii) Bregs-TLR with donor-specific BCR are more suppressive than those derived from B cells with a diverse BCR repertoire (Figure 3); and iv) Bregs-TLR are short-lived and scarce in secondary lymphoid organs, a finding also seen in other reports that further indicate that functional Bregs are dispensable during the maintenance phase of tolerance (17, 23, 24). The possibility that Bregs-TLR would act as antigen-presenting cells to other regulatory cells, including Tregs, is supported by several lines of evidence. Breg-TLR administration was less effective at controlling graft rejection in B cell replete hosts (data not shown), suggesting the involvement of native B cells in graft antigen presentation — implicating MHC molecules — in a tolerogenic manner. However, scRNA-Seq analysis indicated a relative downregulation of MHC class I and class II and BCR expression in Bregs-TLR (Figure 7 and Supplemental Figure 4), discordant with the flow analysis documenting upregulation of these molecules on Bregs-TLR cell surface (Figure 1). Such differences can be attributed to dynamic changes in the activation process of Bregs-TLR, wherein an initial activation and upregulation of MHC and key costimulatory molecules might be followed by downregulation of their transcriptional activity.

When tested both in vitro (Figure 4) and in vivo (Figure 5), Bregs-TLR expressing a donor-specific BCR are always more efficient than polyclonal Bregs at suppressing anti-graft CD4^+^ responses and promoting extended graft survival. Pilot experiments were done to test the hypothesis that Breg activation, through the BCR, is required for optimal suppression. Unfortunately, we had access to a small number of RAG-KO mice that were reconstituted with 5 × 10^6^ OBI Bregs, OTII T cells, transplanted or not, with OVA skin grafts. Preliminary results showed no survival of OBI Bregs in nongrafted recipients even 14 days after injection, a time at which healthy OVA grafts persist on control transplanted mice. This finding would support the view that Breg homeostasis is dependent on BCR sensing of cognate antigen in this OVA model.

scRNA-Seq analyses revealed that the dominant Bregs-TLR cluster 2 had a significant increase in expression of genes involved in the TGF-β pathway (Figure 7, and Supplemental Figure 4). Given that Treg-induction is TGF-β dependent, these data suggest that cluster 2 Bregs-TLR could also mediate suppression through TGF-β. The potential involvement of TGF in Bregs-TLR suppression is comported by an increase in expression of activators of TGF-β in other Breg-TLR clusters, namely, GARP complex subsets (*VPS51*, *VPS52*, and *VPS54*), TGF-β–activated kinase 1 binding proteins (*TAB1*, *TAB2*, and *TAB3*), SMAD-related proteins (*FOS*, *SMAD3*, and *SMAD4*), and integrin subunits (*ITGA4* and *ITGB2*). Of note, the TGF-β pathway is also activated to a lesser extent in Bregs-CpG cells, a likely consequence of activation by CpG (32). Thus, one may consider that other pathways are required to cooperate and/or synergize with TGF-β signaling to achieve optimal Bregs-TLR suppressive functions. We also observed a robust association between the IL-2/STAT5 signaling pathway and functional Breg-TLR cells (Figure 7C, and Supplemental Figure 3B). While IL-2/STAT5 signaling has been shown to be essential for early induction of Foxp3 expression in Tregs (33) as well as for Treg suppressive functions (34, 35), the potential role and function of this pathway in Bregs remains unknown. Further still, the IFN-γ pathway controls, in large part, the activation of MHC class II gene expression on antigen-presenting cells, leading to increased antigen presentation and induction of Th1 tissue-destructive immune responses toward allogeneic transplants (35). In contrast, tolerogenic conditions imposed on B cells, such as treatment with anti-TIM1 mAbs, lessens the production of IFN-γ (11). Our scRNA-Seq data demonstrated a relative enrichment of IFN-γ response in Bregs-CpG compared with Bregs-TLR, further insinuating how Bregs-TLR might prevent allograft rejection by reduction of IFN-γ responses. Collectively, these analyses identify Bregs-TLR cluster 2 as the primary suppressive population, acting through TGF-β and IL-2/STAT5 signaling.

Suppressive Bregs-TLR cells are in fact nonsuppressive Bregs-CpG cells (Figure 2B and Figure 3) that have been further stimulated with the LPI cocktail during the final 5 hours of culture. This short time lapse would not allow detection of transcription factor signatures specific of each subset. Transcriptional factors such as Hivep3, Prdm1, Bcl6, Klf2, Zfp318, or Zfp821 have been reported as critical in B cell differentiation (36). More specifically, B cell–targeted KIF2 deficiency has been associated with dysfunctional mature B cells (36, 37), and selective downregulation of expression in Bregs-TLR (data not shown) places it as a potential Breg lineage determining factor.

A previous study showed that stimulation of naive B cells with high concentrations of CpG induced B cells to differentiate into plasma cells (20). In our current work, we applied doses of CpG approximately 100 times less (1.6 nM vs. 200 nM) to stimulate B cells, and consequently, did not observe the development of CD138^+^ plasma cells (data not shown). Besides Ab production, another property of B cells is to be professional antigen-presenting cells. Following engagement to specific BCR receptors, captured antigens are processed, presented on MHC molecules to T cells, and initiated either tissue-damaging Th1 cells or immunomodulatory T cell responses, according to cues/cytokines from the surrounding milieu (34). In vivo B cell depletion results in a profound decrease in IL-10–producing CD1d^hi^CD5^+^ B10 Bregs (38), increases IFN-γ expression, and renders anti-TIM1 treatment ineffective (11). Thus, it is plausible that some B cell subsets contribute to the induction and/or maintenance of the tolerogenic milieu. Like Tregs, Bregs acquire their suppressive properties from activation via antigen-specific receptors (11, 38), implying that BCR-dependent antigen processing could also be involved in Breg suppression. It is likewise possible that Breg-TLR cells downregulate antigen presentation to effector T cells and/or promote the activation of Tregs in a favorable tolerogenic milieu.

B cell activation can be achieved via BCR, CD40, or TLR (32, 39). Although our work has focused on the Bregs activated by TLR signaling, using a cell line expressing CD40L (39) and a series of cytokines and anti-TIM1, we have also generated B cells with regulatory activity (Bregs-CD40) that also promote graft survival. Interestingly, survival induced by Bregs-CD40 is IL-10 dependent but not dependent on TGF-β. Additional studies examining the regulatory potential of B cells activated via the BCR will help ascertain whether B cell activation by each of these 3 pathways converges on a common outcome of endowing B cell with regulatory properties.

Collectively, our data demonstrate that allograft survival–promoting Bregs can be generated using a protocol that has potential for translation to clinical settings based on its ability to produce large numbers of well-characterized and highly immunosuppressive Bregs. In addition, the increased potency of Bregs carrying a transgenic BCR, as seen in the anti-OVA model, suggests that Breg regulatory functions could be heightened following BCR modifications with chimeric variable fragment receptors for dominant allogeneic determinants, in a manner paralleling that of the CAR technology. Future work to further delineate the mechanisms of action of Bregs induced by dual TLR signaling as well as studies in large animal transplant models can further define the potential of Bregs for clinical translation.

## Methods

### Mice.

WT B6 (C57BL/6, H-2b, and CD45.2), BALB/c (H-2d), B cell–deficient B6 (μMT^–/–^ and H-2b), OVA-transgenic B6 mice (OVA and CD45.2), SCID, OTI (CD45.2), OTII (CD45.2), Pepboy (B6 and CD45.1), and IL-10^–/–^ were purchased from The Jackson Laboratory. CD19cre and TGF-β1flox mice were purchased from The Jackson Laboratory and bred to a genotype of CD19^cre/WT^ TGFβ^fl/fl^, which lacks TGF-β specifically in B cells. OBI mice (B6 background) were provided by Hidde Ploegh at the Whitehead Institute for Biomedical Research, Cambridge, Massachusetts, USA. All mice were housed under specific pathogen–free conditions in the animal facility of Massachusetts General Hospital and used at 6–12 weeks of age.

### In vitro B cell activation.

Spleens were processed to produce single-cell suspensions by manual disaggregation, erythrocyte lysis using an ammonium-chloride-potassium lysing buffer (Gibco), and passage through a 70 μm nylon mesh (Corning). B cells were purified from these single-cell suspensions using the negative isolation EasySep Mouse B Cell Isolation Kit (catalog 19854, STEMCELL Technologies). The purity of B cells following isolation was confirmed via flow cytometry and greater than 97%. Bregs-CpG were produced by culturing purified B cells in complete medium (RPMI 1640 containing 10% fetal bovine serum, 50 μM 2-mercaptoethanol, 1 mM sodium pyruvate, 1× nonessential amino acids, 100 IU/mL penicillin, and 100 μg/mL streptomycin) with CpG B ODN 1668 (10 μg/mL, Class B, murine TLR 9–specific, InvivoGen) for 3 days. Bregs-TLR were generated on Bregs-CpG by adding LPS (10 μg/mL), PMA (50 ng/mL), and ionomycin (1 μg/mL) on the final day for 5 hours before collection. LPS-B cells were cultured with LPS (10 μg/mL) alone for 3 days. All culture additives were purchased from MilliporeSigma unless noted otherwise.

### Cell staining and flow cytometric analysis.

The following anti-mouse Abs were used: CD19 Pacific Blue (clone 6D5, BioLegend), LAP BV421 (clone TW7-16B4, BioLegend), TIM1 PE (clone RMT1-4, BioLegend), CD1d FITC (clone 1B1, BioLegend), CD5 PE-Cy7 (clone 53-7.3, BioLegend), CD25 PerCP-Cy5.5 (clone PC61, BioLegend), CD9 AF647 (clone MZ 3, BioLegend), PD-L1 PE-Cy7 (clone 10F.92G, BioLegend), IgM PE (clone R6-60.2 BD Biosciences), IgD APC (clone 11-26c.2a, BioLegend), IgG FITC (clone 11-4011-85, eBioscience), CD21/35 FITC (clone eBio4E3, eBioscience), CD23 PE-Cy7 (clone B3B4, BioLegend), CD80 PE (clone 16-10A1, BioLegend), CD86 FITC (clone GL-1, BioLegend), OvalbuminFlex488 (clone O34781, Invitrogen), CD4 PE-Cy7 (clone GK1.5, BioLegend), CD8 PE (clone 53-6.7, BioLegend), TCR Vα2 PerCP-Cy5.5 (clone B20.1, BioLegend), TCR Vβ5 PE (clone MR-9-4, BioLegend), and CD45.2 eFluor 450 (clone 104, eBioscience). Cultured B cells were first stained with Fixable Viability Dye eFluor 780 (catalog 65-0865-14, eBioscience) for 20 minutes at 4°C, washed, and then stained with surface markers at 4°C for 30 minutes. For intracellular staining, cells were fixed and permeabilized using a fixation/permeabilization kit (catalog 00-5523-00, eBioscience). Samples were then intracellularly stained with Abs against Foxp3 APC or PerCP-Cy5.5 (clone FJK16s, Invitrogen). For cytokine detection, cells were stimulated by adding LPS (10 μg/mL), PMA (20 ng/mL), ionomycin (1 μg/mL), and GolgiStop Protein Transport Inhibitor (catalog 51-2-92KZ, BD Biosciences) for 5 hours; fixed; and permeabilized. Samples were then intracellularly stained with IL-10 APC or FITC (clone JES5-16E3, BioLegend), IL-17a PerCP-Cy5.5 or PE (clone TC11-18H10.1, BioLegend), IL-4 PE (clone 11B11, BioLegend), or IFN-γ APC (clone XMG1.2, BioLegend). All samples were treated with 1 μL of Fc-block (CD16/CD32, clone 93, BioLegend) before staining. Samples were run on a BD FACSVerse flow cytometer (Becton Dickinson) and analyzed using FlowJo v10 analysis software (BD).

### In vitro suppression assay.

Responder T cells were purified from B6 spleens using an EasySep Mouse T Cell Isolation Kit (catalog 19851, STEMCELL Technologies) and labeled with CellTrace Violet Cell Proliferation Kit (catalog C34557, Invitrogen). A total of 1.5 × 10^5^ CTV-labeled T cells were cocultured with 3 × 10^5^ activated or unactivated syngeneic B cells and with CD3/CD28 Dynabeads (catalog 11456D, Gibco). On day 4, cells were analyzed by flow cytometry for proliferation. For the dose-dependent assay, B cells were added in varying amounts, such as 4.5 × 10^5^, 3 × 10^5^, 1.5 × 10^5^, and 0.75 × 10^5^.

### Islet transplantation and adoptive transfer experiments.

Diabetes in B6 μMT^−/−^ mice was induced by a single i.p. injection of 200 mg/kg streptozotocin (catalog S0130, MilliporeSigma). Diabetes was defined as blood glucose levels greater than 300 mg/dL for at least 2 consecutive days. Islets from BALB/c donors were isolated by the standard technique of collagenase digestion and Ficoll density gradient purification. Approximately 500 fresh islets were transplanted under the kidney capsule of diabetic mice. Euglycemia was defined as a nonfasting blood glucose level of less than 200 mg/dL. Graft rejection was diagnosed when mice developed hyperglycemia again, with blood glucose greater than 200 mg/dL for at least 2 consecutive readings. Allograft function was confirmed by nephrectomy of the kidney containing the transplanted islets on day 100 after transplantation. All recipients with long-term grafts became hyperglycemic within 48 hours of nephrectomy. For adoptive transfer studies, 5 × 10^6^ naive or activated B cells were administered i.v. into islet recipients 1 day before transplantation.

### Skin transplantation and adoptive transfer experiments.

Skin graft transplantation was conducted to follow the technique of Billingham and Medawar as previously described (40). The donor animal was euthanized and hair-clipped; the full-thickness skin graft was harvested and processed, then cut into 1.2 cm × 1.2 cm squares and kept in PBS on ice. After the donor skin preparation, the recipient was anesthetized and shaved, and a square of recipient skin was removed to match the donor graft. Donor skin was attached with collodion and secured with dressing and skin staples. After 10 days, the dressing was removed. Graft rejection was designated if the skin graft contracted to less than 10% of the original size. For adoptive transfer studies, 5 × 10^6^ naive or activated B cells were administered i.v. into skin recipients.

### In vivo suppression assay.

CD45.1 B6 mice received 3, 5, or 10 × 10^6^ Bregs-TLR from B6 or OBI, or 5 × 10^6^ naive B cells from OBI or B6 1 day before OVA skin transplantation. On day 7, 1 × 10^6^ CFSE-labeled OTII or CTV-labeled OTI T cells were i.v. injected. On day 10, the DLNs, N-DLNs, and spleens were harvested and processed as the single-cell suspensions to determine T cell proliferation by FACS.

### Immunotherapy and adoptive transfer for regulatory T cell induction.

For Breg-Treg Foxp3 induction, naive CD4^+^CD25^–^ OTII T cells were sorted by a FACSAria (BD Biosciences) Cell Sorter and adoptively transferred into naive SCID mice that received 1 × 10^6^ CD4^+^CD25^–^ OTII T cells and 5 × 10^6^ activated B cells from B6 or OBI by i.v. injection on day 0. At the same time, SCID mice were transplanted with OVA skin and received 200 μg anti–TGF-β Ab (clone 1D11.16.8, Bio X Cell), 250 μg anti–IL-10 Ab (clone JES5-2A5, Bio X Cell), or 200 μg mouse IgG1 isotype control (clone MOPC-21, Bio X Cell) on days 0, 2, 4, 6, and 8 after transplant. On day 14, the DLNs of the mice were harvested, processed, and stained for Foxp3 expression on CD4^+^CD25^+^ T cells.

### scRNA-Seq and data processing.

scRNA-Seq data were processed using the 10X Genomics platform at the Department of Molecular Biology at Massachusetts General Hospital. For each sample, an estimated 5000 cells were used for Gelbeads-in-Emulsion (GEM) generation and libraries were prepared using the recommended protocol (Chromium Single Cell 3’ v3). Fastq files were demultiplexed using Illumina bcl2fastq, followed by alignment, filtering, barcode counting, and unique molecular identifier counting using CellRanger version 3.1.0. As a reference, mouse mm10 (Ensembl 93) version 3.0.0 and CellRanger aggr were used to combine and normalize GEM wells. All data are available in the Gene Expression Omnibus (GEO number: GSE208527). To prevent sparse and noisy scRNA-Seq data from hindering downstream analysis, SAVER-X was used for gene expression denoising and imputation (41). After inputting the data in Seurat, high-quality cells with 1,000 expressed genes and less than 10% mitochondrial reads were selected (42). Standard procedures for dimensionality reduction were followed (principal component analysis, t-distributed stochastic neighbor embedding, and uniform manifold approximation and projection). A shared nearest neighbor modularity optimization-based clustering algorithm was performed with different “findCluster” resolutions (0.1, 0.3, and 0.5) to identify cell clusters. The MAST package was utilized to identify differentially expressed genes (DEGs) between clusters and annotate the subsets (43) and the markers for each cell subset. We created specific clusters based on DEGs. HALLMARK, C2, and C5 databases were used for downstream gene enrichment analysis (44). ComplexHeatmap package was used for the result display (45).

### Statistics.

Statistical analysis was performed using GraphPad Prism (version 9.0.2, GraphPad Software). Graft survival between experimental groups was compared using Kaplan-Meier survival curves and significance was assessed by a log-rank test. Differences between experimental groups were analyzed using a 2-tailed unpaired Student’s *t* test (for parametric data) or a Mann-Whitney/Kruskal-Wallis test (for nonparametric data). Other differences among more than 2 groups were analyzed using 1-way ANOVA (for parametric data) or Kruskal-Wallis test (for nonparametric data). A *P* value less than 0.05 was considered statistically significant: *P < 0.05; **P < 0.01; ***P < 0.001; ****P < 0.0001.

### Study approval.

All protocols were performed following the principles of laboratory animal care and approved by the Institutional Animal Care and Use Committee at Massachusetts General Hospital.

## Author contributions

KML, QF, GH, and LK designed and performed experiments, analyzed data, generated figures, and wrote the manuscript. KD and LW provided technical help and reagents. QF, DA, and PVG analyzed RNA-Seq data. JL, SK, NT, AN, and HY provided data analysis and critical reading of the manuscript. CL and CGR assisted with the editing and writing of the manuscript. JFM designed and supervised the study and wrote and edited the manuscript.

## Supplementary Material

Supplemental data

## Version 1 08/09/2022

In-Press Preview

## Version 2 09/08/2022

Electronic publication
