# Supplementary material for: Suppression of allograft rejection by regulatory B cells induced via TLR signaling
Source: JCI Insight. 2022 Sep 8;7(17):e152213. doi: 10.1172/jci.insight.152213 (PMC9536278; doi:10.1172/jci.insight.152213)
Supplement: Supplemental data [file jciinsight-7-152213-s182.pdf]

Supplementary Figures

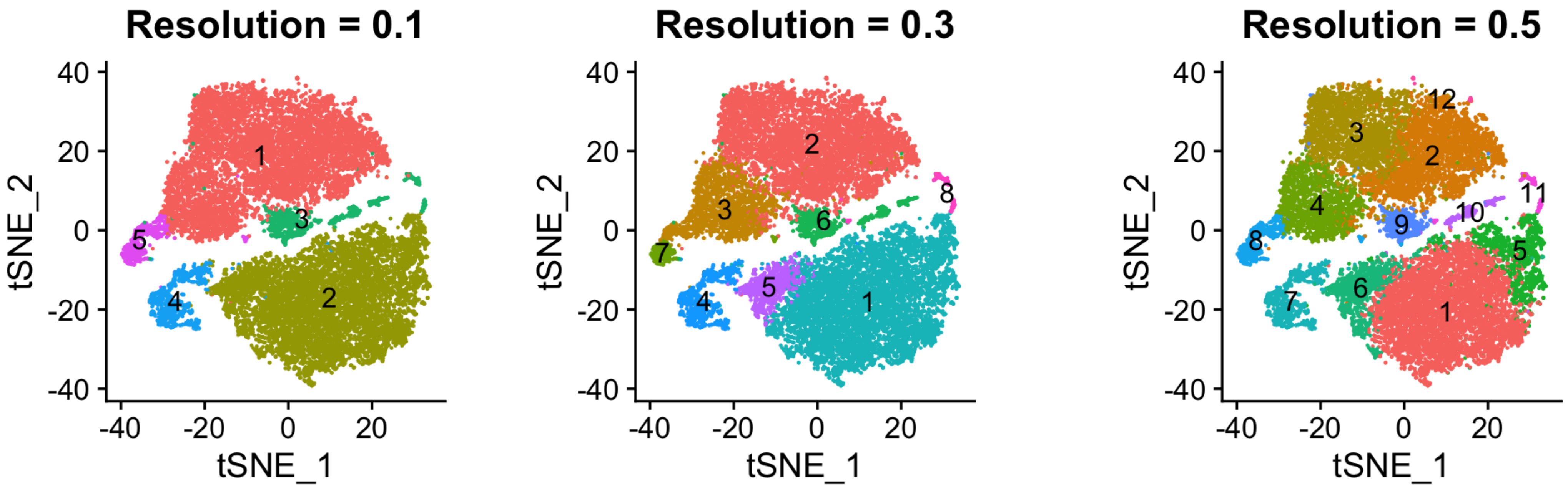

Supplementary Figure S1. Cluster analysis in Bregs-TLR and Bregs-CpG cells by scRNA-seq. Cluster analysis strategy using different resolution values. For cluster analysis, when the parameter "resolution" varied, the cluster numbers differed. When the parameter resolution was 0.5, there were 12 cell clusters in total. However, there were no differentially expressed genes (DEGs) (log|FC| >1 and $p < 0.05$) found among some subsets of Bregs-CpG (such as clusters 2, 3, and 12). We used the clustering result in which the resolution was 0.3 for subsequent analysis.

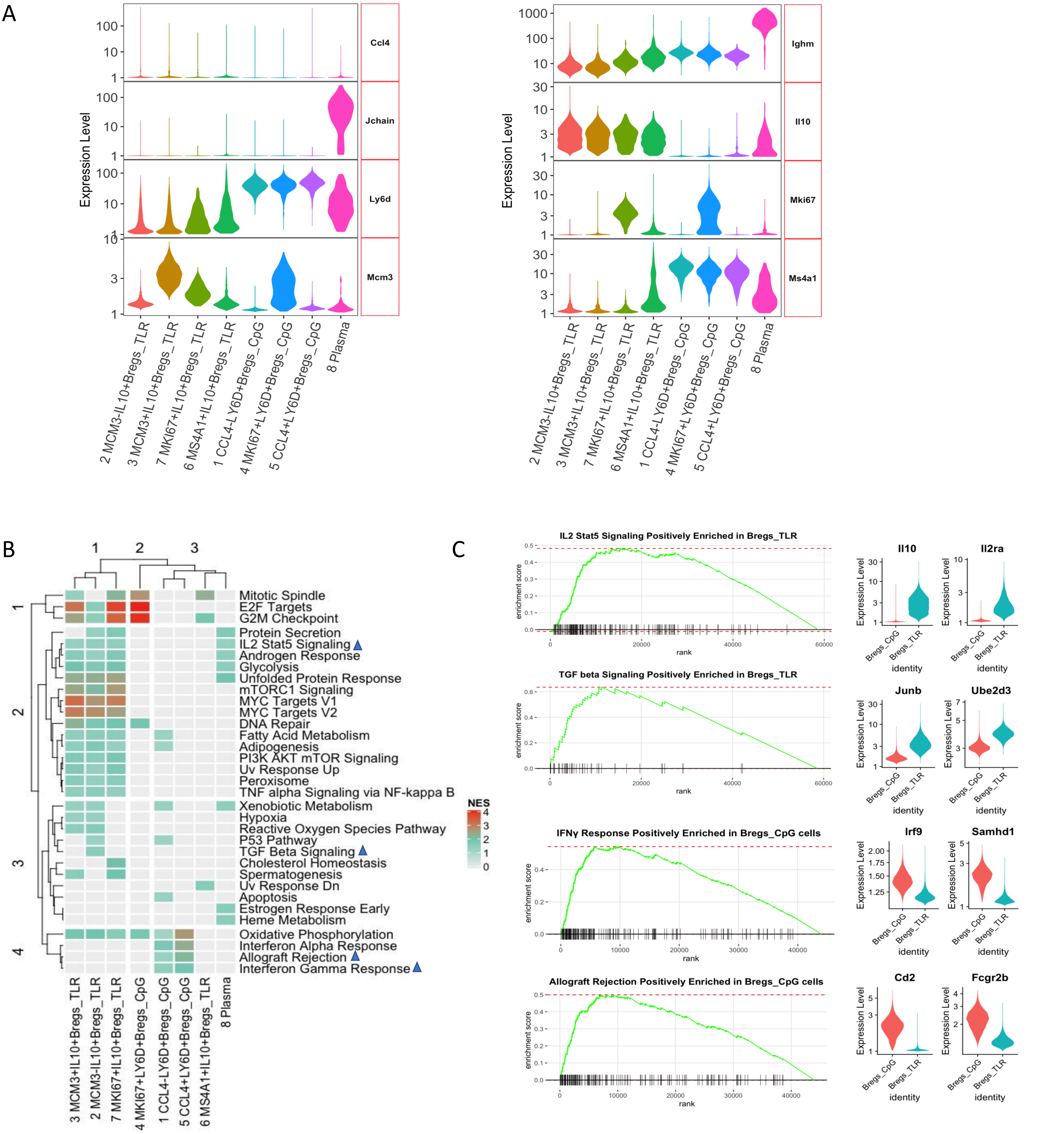

Supplementary Figure S2. Annotation and Pathway enrichment analysis of Bregs-TLR by scRNA-seq. **A** Annotation markers of Bregs-TLR and Bregs-CpG cells. **B** Pathway enrichment analysis in each cluster by scRNA-seq using HALLMARK database. **C** IL2 Stat5 signaling, and TGF-beta signaling were positively enriched in Bregs-TLR, while IFNγ response, and allograft rejection were positively enriched in Bregs-CpG cells.

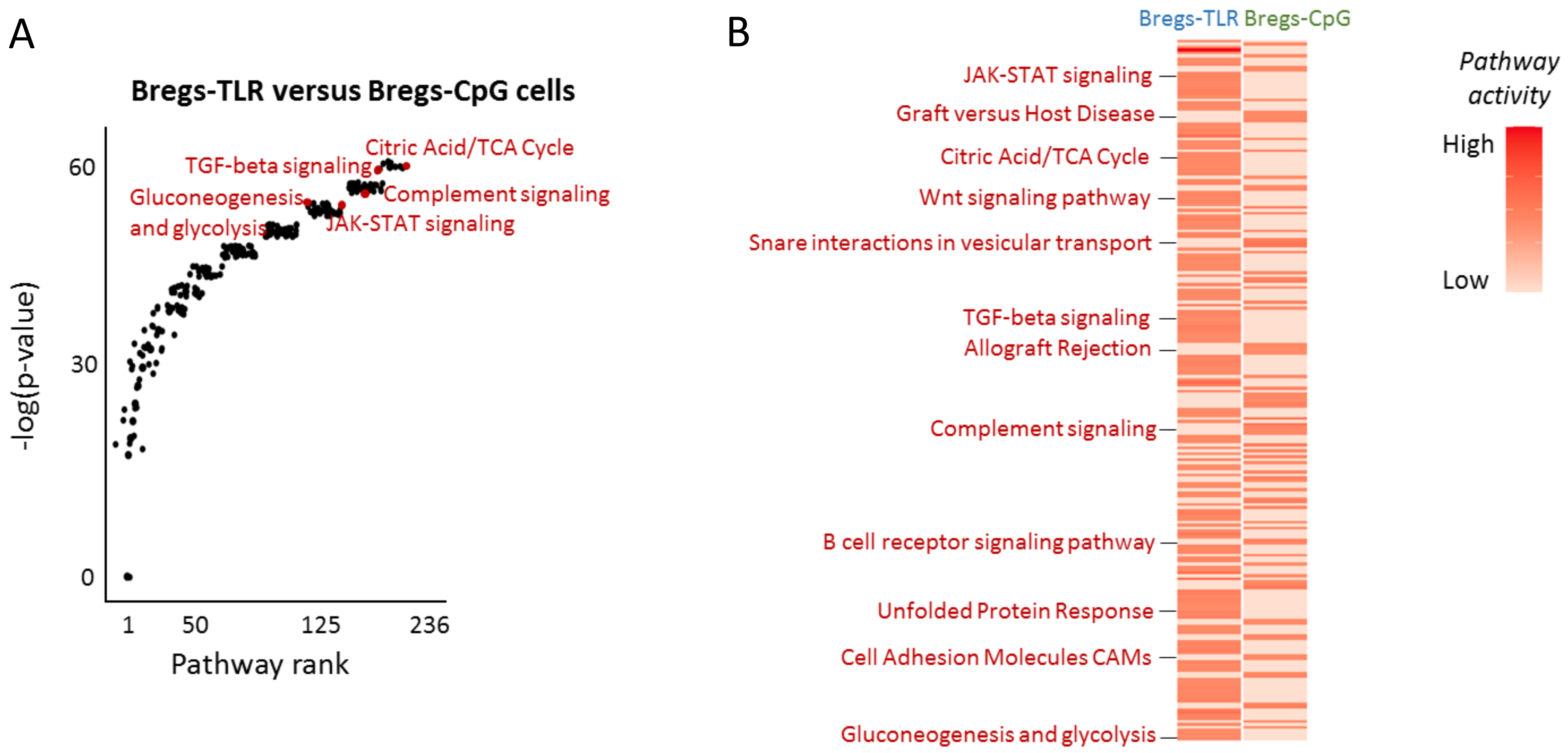

Supplementary Figure S3. Pathway enrichment analysis using KEGG and HALLMARK databases. A Pathways enriched between Bregs-TLR and Bregs-CpG. The pathways were ordered according to the –log(p-value) and the top several pathways were labelled in the plots. **B** Heatmap of enriched pathways in TLR-Bregs and Bregs-CpG.

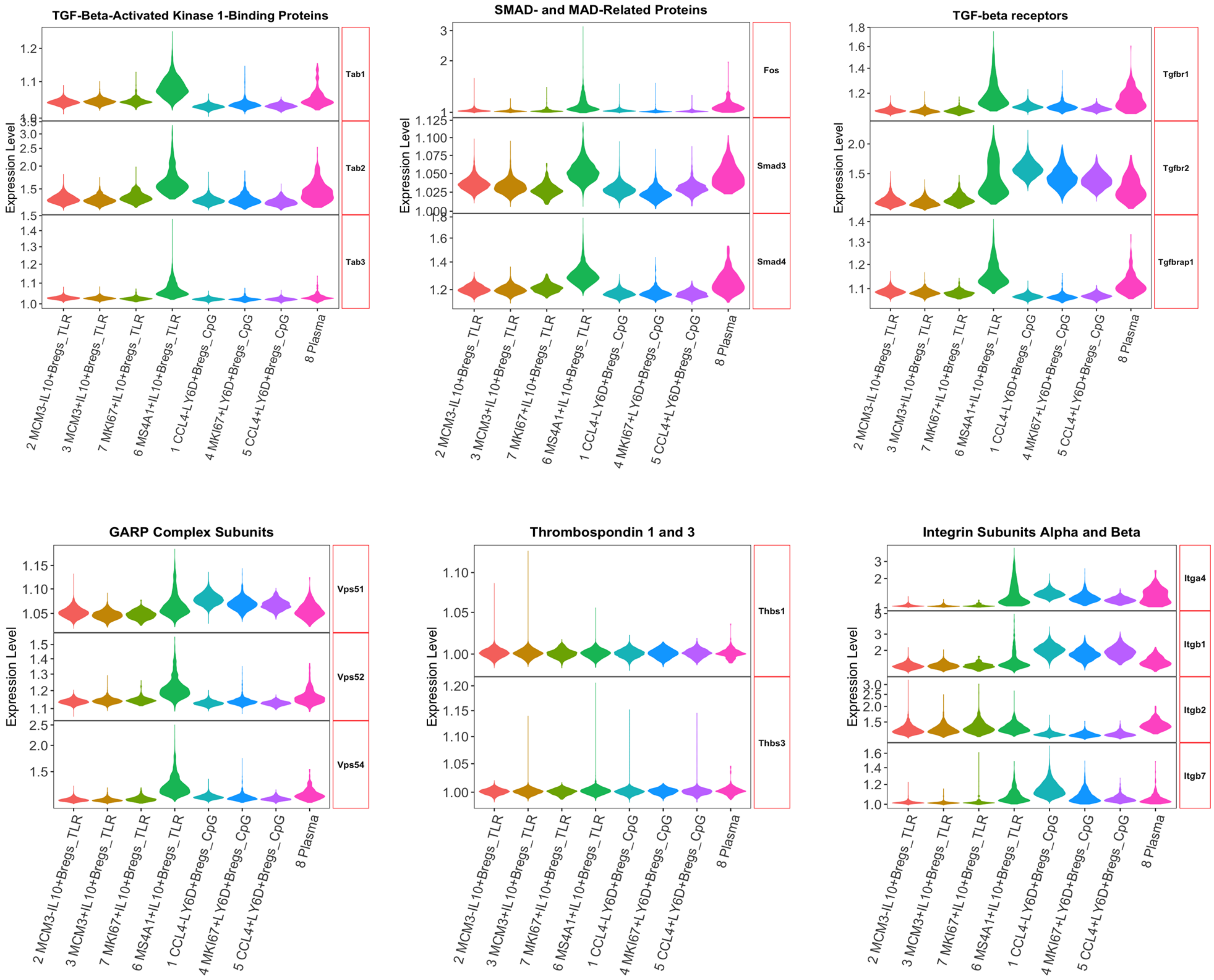

Supplementary Figure S4. TGF-beta activation related transcriptional factors and proteins expression.

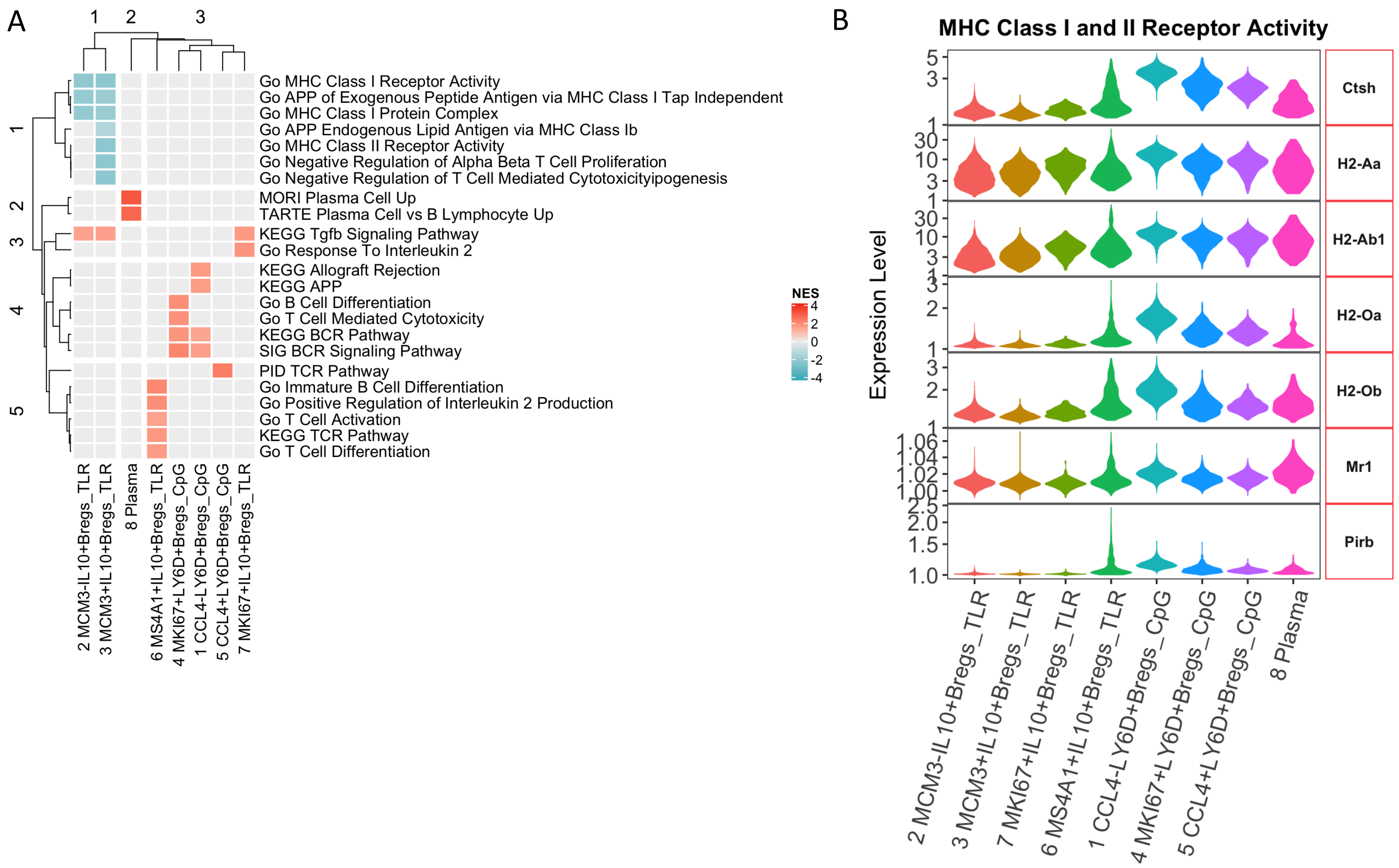

Supplementary Figure S5. Pathway enrichment analysis using C2/C5 databases. A Pathway enrichment analysis in each cluster by scRNA-seq using C2/C5 database. MHC-relevant, BCR, antigen processing and presentation (APP), and TCR signaling were displayed in the heatmap. **B** Vlnplot of the Genes enriched in MHC class II receptor activity. Those genes were upregulated in clusters 1, 4, 5, and 6 while downregulated in clusters 2, 3, and 7.

Note : C5 refers to GO enrichment analysis, C2 refers to KEGG and other datebases.
See in details: https://www.gsea-msigdb.org/gsea/index.jsp
